# Trends in lung cancer mortality in South Africa: 1995-2006

**DOI:** 10.1186/1471-2458-11-209

**Published:** 2011-04-04

**Authors:** Braimoh Bello, Olufolawajimi Fadahun, Danuta Kielkowski, Gill Nelson

**Affiliations:** 1 Reproductive Health and HIV Research Unit, Faculty of Health Sciences, University of the Witwatersrand, Johannesburg, South Africa; 2 National Institute for Occupational Health, National Health Laboratory Service, Johannesburg, South Africa; 3 School of Public Health, Faculty of Health Sciences, University of the Witwatersrand, Johannesburg, South Africa

## Abstract

**Background:**

Cancer remains a major cause of morbidity and mortality worldwide. In developing countries, data on lung cancer mortality are scarce.

**Methods:**

Using South Africa's annual mortality and population estimates data, we calculated lung cancer age-standardised mortality rates for the period 1995 to 2006. The WHO world standard population was used as the reference population. Scatter plots and regression models were used to assess linear trends in mortality rates. To better characterise emerging trends, regression models were also partitioned for defined periods.

**Results:**

Lung cancer caused 52,217 deaths during the study period. There were 4,525 deaths for the most recent year (2006), with men accounting for 67% of deaths. For the entire South African population, the age-standardised mortality rate of 24.3 per 100,000 persons in 1995 was similar to the rate of 23.8 per 100,000 persons in 2006. Overall, there was no significant decline in lung cancer mortality in South Africa from 1995 to 2006 (slope = -0.15, p = 0.923). In men, there was a statistically non-significant annual decline of 0.21 deaths per 100,000 persons (p = 0.433) for the study period. However, from 2001 to 2006, the annual decline of 1.29 deaths per 100,000 persons was statistically significant (p = 0.009). In women, the mortality rate increased significantly at an annual rate of 0.19 per 100,000 persons (p = 0.043) for the study period, and at a higher rate of 0.34 per 100,000 persons (p = 0.007) from 1999 to 2006.

**Conclusion:**

The more recent declining lung cancer mortality rate in men is welcome but the increasing rate in women is a public health concern that warrants intervention. Smoking intervention policies and programmes need to be strengthened to further reduce lung cancer mortality in men and to address the increasing rates in women.

## Background

Cancer is a major cause of morbidity and mortality, worldwide. The World Health Organisation (WHO) estimates show that the global burden of cancer is increasing, with new cases expected to rise by 50% over the next 20 years [1]. Research published in 2010 estimated that, globally, there were 12.7 million new cases of cancer with 7.6 million deaths in 2008; 56% of new cases and 63% of cancer deaths occurred in developing countries [2]. The emergence of cancer morbidity and the increase in mortality in South Africa are well documented and have been attributed to different factors, including smoking, occupational exposures, infections, changing lifestyles, and environmental pollutants [1,3,4]. In 2003, the South African Medical Research Council published burden of disease estimates and listed cancer as the fourth leading cause of death and the eighth major contributor to disability adjusted life years (DALYs) in the country [5].

Lung cancer is most commonly attributed to smoking; 80-90% of lung cancer cases are attributed to smoking and a smaller proportion (10-20%) is attributed to occupational exposure to agents such as uranium, ionising radiation, asbestos, silica, arsenic, beryllium, chloromethyl, nickel chromates, indoor emissions from burning fuels, and polycyclic aromatic hydrocarbons (PAHs) [6-8]. The acquired immunodeficiency syndrome (HIV/AIDS) has also recently been associated with the development of lung cancer. A study on a cohort of HIV positive individuals on antiretroviral therapy (ART) showed that surviving individuals have an increased risk of cancers previously not associated with HIV, such as lung cancer, neck and head cancers, liver cancer and rare anal cancer [9].

Recent changes in smoking prevalence have produced changes in lung cancer incidence and mortality worldwide. While there has been a substantial decline in lung cancer rates in developed countries, such as the United States of America (USA), Canada and many parts of Europe [10-13], incidence rates are reportedly rising in newly industrialised and developing countries like China and India [14]. There are observed gender differences in these rates, partly due to the delay in the uptake of smoking among women, which usually lags behind that of men by approximately 25 years [15-17]. This is reflected in the increasing lung cancer mortality rates seen in women, while the rates in men have leveled off or are decreasing in many parts of the world [10-13,17].

An electronic search of the published literature revealed that the most recent study on lung cancer mortality trends in South Africa was published in 1985, covering the period 1949 to 1979 [18]. The results showed an increasing trend in rates for lung cancers in men. In contrast, rates were much lower and more stable in women. No more recent data on cancer trends have been published.

The aim of this study was to determine trends in lung cancer mortality in the South African general population for the period 1995 to 2006. Results are presented for the entire population and by gender. The study also sought to better characterise emerging trends by carrying out gender-specific partitioned analyses for short-term trends. The results will help to understand the direction of the lung cancer epidemic in South Africa and can assist in monitoring and projecting future rates. This will help to inform the need for public health interventions. The results may also be useful to health practitioners and policy makers in other developing countries.

## Methods

### Cancer deaths and population estimates

Both numerator (lung cancer deaths) and denominator (estimates of populations at risk) data were obtained from Statistics South Africa (StatsSA). StatsSA is the South African government body responsible for the collection, production and dissemination of official and other statistics, the conduct of population census, and the coordination of statistics produced by other organisations in the country.

Anonymous raw mortality data were obtained from StatsSA, from which numbers of deaths due to lung cancer were extracted. Lung cancer deaths were defined as deaths due to cancers of the lung and bronchus reported on death certificates. The underlying cause of death was coded by StatsSA. For the period 1996 to 2006, the 10^th ^International Classification of Diseases (ICD 10) codes [19] were used, while ICD 9 codes [20] were used for the year 1995. For the denominator, mid-year total population estimates by age group and gender were available for the entire study period. However, disaggregated population estimates by age group were unavailable for the years 1995, and 1997 to 2000. The population distribution, by age group, for the year 2001 (census year) was therefore applied to the population estimates to obtain disaggregated population figures by age group for those years.

### Data analysis

Data received from StatsSA were in text and Microsoft Excel formats and were converted to STATA 10 format for statistical analysis (StataCorp, 2008 Texas, USA). For descriptive purposes, the numbers of deaths due to lung cancer were reported by gender and age group for the most recent year, 2006. To estimate age-standardised mortality rates for each year, the WHO world standard population structure was used as the reference population. Age-standardised rates were reported per 100,000 persons.

For trend analyses, scatter plots (presented as line graphs) of age-standardised rates against year of death were plotted, and regression models were fitted to assess linear trends in the age-standardised mortality rates. Models were fitted for the entire study period, and were also partitioned from 2001 to 2006 for men and from 1999 to 2006 for women. Ninety five percent confidence intervals (95% CI) were calculated for regression slopes. Statistically significant slopes (p-values < 0.05) were interpreted as average annual increases/decreases in age-standardised mortality for the period.

## Results

### Deaths due to lung cancer

During the study period, there were 52,217 deaths due to lung cancer. In the most recent year (2006), there were 4,525 deaths. There were marked differences in the numbers of deaths by gender and age. Most deaths (67.4%) occurred in men. The majority of cases were 50 to 69 years of age (Table 1).

**Table 1:** Numbers of deaths due to lung cancer in South Africa for 2006 by gender and age group

*Variable*	*Lung cancer* *n (%)*
Gender	
Men	3049 (67.4)
Women	1476 (32.6)

Age group	
20 - 29	22 (0.5)
30 - 39	94 (2.1)
40 - 49	464 (10.3)
50 - 59	1135 (25.1)
60 - 69	1420 (31.4)
70 - 79	980 (21.7)
80 and older	410 (9.1)

### Trends in mortality due to lung cancer

For the entire South African population, the age-standardised mortality rate of 24.3 per 100,000 persons in 1995 was similar to the rate of 23.8 per 100,000 persons in 2006. Overall, there was no significant decline in the annual lung cancer mortality rate from 1995 to 2006 (slope = -0.15, p = 0.923). However, the rate for men decreased from 44.2 per 100,000 persons in 1995 to 39.4 per 100,000 persons in 2006, while the rate for women increased from 10.8 to 13.4 per 100,000 persons (Table 2).

**Table 2:** Annual deaths and age-standardised mortality rates due to lung cancer in South Africa from 1995 to 2006, by gender

*Year*	*Men*		*Women*	
	
	*No. of deaths*	Rate*	*No. of deaths*	Rate*
1995	3019	44.2	1048	10.8
1996	3281	49.5	1293	13.9
1997	2869	40.3	1124	11.0
1998	3038	41.5	1223	11.7
1999	3184	42.8	1209	11.3
2000	2975	39.8	1195	11.1
2001	3121	46.6	1242	13.1
2002	3089	44.8	1217	12.4
2003	3038	45.1	1288	12.8
2004	3233	44.7	1413	13.5
2005	3153	41.9	1440	13.5
2006	3049	39.4	1476	13.4

* Rates per 100,000 persons are standardised to the WHO world standard population.

**Figure 1:**
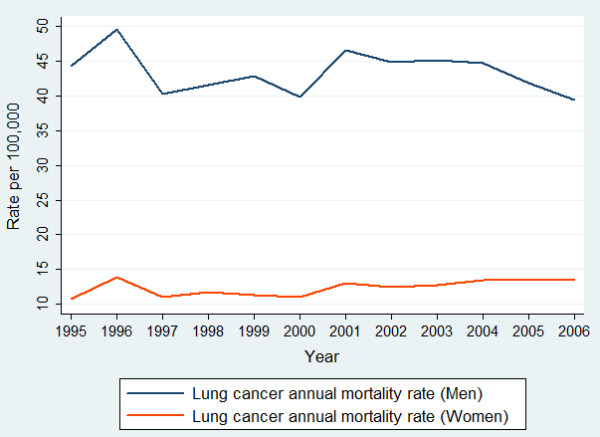
**Trends in age-standardised mortality rates (per 100,000 persons) for lung cancer in South Africa from 1995 to 2006, by gender**.

Figure 1 shows that, for men, the rates of lung cancer mortality decreased from 2001 whereas, for women, the rates increased from 1999. For this reason, in addition to fitting a linear regression model for the entire study period, models were also fitted from 2001 to 2006 for men and from 1999 to 2006 for women.

The trends in men showed a statistically non-significant annual decline of 0.21 deaths per 100,000 persons for the study period. However, when the analysis was partitioned for short-term trends, a statistically significant average annual decline of 1.29 deaths per 100,000 persons was observed from 2001 to 2006. In women, a significant annual increase of 0.19 per 100,000 persons was observed for the study period. This increase was more pronounced from 1999 to 2006 at 0.34 deaths per 100,000 persons (Table 3).

**Table 3:** Regression coefficients for linear trends in age-standardised mortality rates of lung cancer in South Africa from 1995 to 2006

*Group*	*1995 to 2006*		*Partitioned analysis*		
	
	*Slope (95% CI)*	*P-value*	*Period*	*Slope (95% CI)*	*P-value*
Entire population	-0.15 (-0.34 to 0.32)	0.923			
Men	-0. 21 (-78 to 0.36)	0.433	2001- 2006	-1.29 (-2.0 to -0.53)	0.009
Women	0.19 (0.01 to 0.37)	0.043	1999 - 2006	0.34 (0.13 to 0.54)	0.007

## Discussion

A previous cancer trend study covering the period 1949 to 1979 demonstrated increasing lung cancer mortality for men in South Africa [18]. Our results present a more recent trend. The lung cancer mortality rate for men remained stable from 1995 to 2000 but declined significantly from 2001 to 2006, by approximately 1.3 deaths per 100,000 persons (p < 0.05) per year. Assuming that this trend continues, the mortality rate of 39.4 deaths per 100,000 persons in 2006 would decrease by one-third in approximately one decade.

While mortality due to lung cancers is on the decline for men, the trends are in the opposite direction for women. The rate of lung cancer mortality in South African women was relatively low and stable until 1979 [18]. The recent trend shows that the age-standardised mortality rate increased by 24.1% from 1995 to 2006, at an average annual rate of 0.19 deaths per 100,000 persons. In the most recent years (1999 to 2006), the rate increased even faster at 0.34 deaths per 100,000 persons. Assuming that this increase continues for the next decade, the mortality rate of 13.4 per 100,000 in 2006 will reach 16.8 per 100,000 by 2016. As in many countries, South Africa is probably yet to experience the full impact of smoking on the incidence of tobacco-related cancers in women.

Declining trends in lung cancer mortality rates for men and increasing trends for women have also been reported in Europe and America [10-13,15-17,21-23]. In the United Kingdom, for example, age-standardised lung cancer mortality in men decreased from 108 per 100,000 persons in 1978 to 52 per 100,000 persons in 2007 [22]. In Italy, mortality due to lung cancer and other tobacco-related cancers was reportedly declining in men but not in women [12]. Data presented for the entire Europe showed that, overall, female lung cancer mortality increased from 5.5 per 100,000 in 1965 to 11.2 per 100,000 in 2001 [17]. In fact, the lung cancer mortality rate of 13.1 per 100,000 found in our study for 2001, is similar to the 11.2 per 100,000 persons reported for the same year in Europe [17]. Recent American studies have also reported that lung cancer mortality has declined in men but not in women [11,13,23].

The decline in lung cancer mortality in men in South Africa may be attributed to health promotion programmes, including public enlightenment campaigns and the government's comprehensive anti-smoking policies. These policies include consistent increases in the tobacco excise tax (and, therefore, the cost of cigarettes) and the ban on public smoking. These public health interventions have produced a consistent decline in smoking prevalence amongst men in South Africa [24]. Van Walbeek reported that aggregate cigarette consumption dropped by 26% from 1993 to 2000 [25], 60% of which could be explained by a reduction in the average number of cigarette smoked, and the remaining 40% by a reduction in smoking prevalence [25]. Cheyip *et al. *reported a significant decline in smoking prevalence among men employed by a South African platinum mining company [26].

While public health interventions, initiated more than two decades ago, have contributed to the decline in lung cancer mortality, the impact of the reduction in cigarette smoking may become more obvious in the next decades, given the long latency period of lung cancer. The effect of these interventions was observed in men and not women because of the historical difference in smoking prevalence by gender [15,24,25].

Another factor that may affect the lung cancer mortality rate is the impact of competing causes of death due to the HIV/AIDS epidemic. Since 1997, HIV-related deaths have caused a shift in adult mortality towards communicable diseases [27] and have also shortened life expectancy from 63 years in 1990 to 51 years in 2006 [28]. This, in turn, has reduced the probability of the development of cancers with long latency periods, such as lung cancer, and has thus caused a reduction in lung cancer incidence.

Also of note are reports of changing patterns of cancers due to anti-retroviral treatment (ART), with increased occurrence of cancers that are not traditionally linked to suppressed immunity, such as lung cancer [9,29,30]. How ART increases the likelihood of such cancers, and the duration of therapy required for them to develop, is still unknown. The influence of ART on South African lung cancer mortality trends remains to be seen as rollout of ART began only in 2003 [31].

## Conclusions

The trends in lung cancer mortality in South Africa are similar to those in developed countries. Smoking intervention policies and programmes need to be strengthened to further reduce lung cancer mortality in men and to address the increasing rates of lung cancer in women.

## Competing interests

The authors declare that they have no competing interests.

## Authors' contributions

BB substantially contributed to the conception and design of the study, literature review, analysis and interpretation of data, drafted the manuscript and approved the final version. OF carried out statistical analyses and interpretation of data, contributed to drafting and revising the manuscript and approved the final version. DK contributed to the conception and design of the study, sought data from Statistics South Africa, contributed to the literature review, analysis and interpretation of data, drafted the manuscript, and approved the final version. GN contributed to the conception and design of the study, interpretation of data, and drafting of the manuscript, and approved the final version. All authors read and approved the final manuscript.

## Authors' information

Braimoh Bello, MSc; M.Sc (Med) Medical Epidemiologist: Reproductive Health and HIV Research Unit Faculty of Health Sciences, University of the Witwatersrand, Johannesburg South Africa

Olufolawajimi Fadahun, MD Medical Scientist: Epidemiology and Surveillance Unit National Institute for Occupational Health, National Health Laboratory Service South Africa

Danuta Kielkowski, PhD Head: Epidemiology and Surveillance Unit National Institute for Occupational Health, National Health Laboratory Service South Africa

Gill Nelson, MSc (Med) Research Scientist: Pathology Division National Institute for Occupational Health, National Health Laboratory Service South Africa

## Pre-publication history

The pre-publication history for this paper can be accessed here:

http://www.biomedcentral.com/1471-2458/11/209/prepub
